# Comparing CB1 receptor GIRK channel responses to receptor internalization using a kinetic imaging assay

**DOI:** 10.1038/s41598-024-68451-2

**Published:** 2024-08-07

**Authors:** Haley K. Andersen, Duncan G. Vardakas, Julie A. Lamothe, Tannis E. A. Perault, Kenneth B. Walsh, Robert B. Laprairie

**Affiliations:** 1 College of Pharmacy and Nutrition, University of Saskatchewan, Saskatoon, SK Canada; 2 Pharmacology, Physiology, and Neuroscience, School of Medicine Columbia, University of South Carolina, Columbia, SC USA; 3 Department of Pharmacology, College of Medicine, Dalhousie University, Halifax, NS Canada

**Keywords:** Cannabinoid receptor, Cannabinoid, G protein-coupled receptor, Receptor trafficking, Receptor signaling, Real-time assay, Pharmacology, Molecular neuroscience

## Abstract

The type 1 cannabinoid receptor (CB1R) mediates neurotransmitter release and synaptic plasticity in the central nervous system. Endogenous, plant-derived, synthetic cannabinoids bind to CB1R, initiating the inhibitory G-protein (G_i_) and the β-arrestin signaling pathways. Within the G_i_ signaling pathway, CB1R activates G protein-gated, inwardly-rectifying potassium (GIRK) channels. The β-arrestin pathway reduces CB1R expression on the cell surface through receptor internalization. Because of their association with analgesia and drug tolerance, GIRK channels and receptor internalization are of interest to the development of pharmaceuticals. This research used immortalized mouse pituitary gland cells transduced with a pH-sensitive, fluorescently-tagged human CB1R (AtT20-SEPCB1) to measure GIRK channel activity and CB1R internalization. Cannabinoid-induced GIRK channel activity is measured by using a fluorescent membrane-potential sensitive dye. We developed a kinetic imaging assay that visualizes and measures CB1R internalization. All cannabinoids stimulated a GIRK channel response with a rank order potency of WIN55,212-2 > (±)CP55,940 > Δ^9^-THC > AEA. Efficacy was expressed relative to (±)CP55,940 with a rank order efficacy of (±)CP55,940 > WIN55, 212-2 > AEA > Δ^9^-THC. All cannabinoids stimulated CB1R internalization with a rank order potency of (±)CP55,940 > WIN55, 212-2 > AEA > Δ^9^-THC. Internalization efficacy was normalized to (±)CP55,940 with a rank order efficacy of WIN55,212-2 > AEA > (±)CP55,940 > Δ^9^-THC. (±)CP55,940 was significantly more potent and efficacious than AEA and Δ^9^-THC at stimulating a GIRK channel response; no significant differences between potency and efficacy were observed with CB1R internalization. No significant differences were found when comparing a cannabinoid’s GIRK channel and CB1R internalization response. In conclusion, AtT20-SEPCB1 cells can be used to assess cannabinoid-induced CB1R internalization. While cannabinoids display differential G_i_ signaling when compared to each other, this did not extend to CB1R internalization.

## Introduction

Cannabinoid receptors have gained interest due to their potential in a range of therapeutic applications, such as anxiety, depression, obesity, pain, and neurodegenerative disorders^1,2^. The endocannabinoid system canonically consists of two G protein-coupled receptors (GPCR), cannabinoid-type 1 (CB1R) and cannabinoid-type 2 (CBR2)^3^. CB1R is predominantly expressed in the central nervous system (CNS), where it modulates neuronal activity through the inhibitory G protein signaling complex (Gαβγ_i_) and β-arrestin signaling^1,4,5^. These signaling pathways have gained notoriety as the same pathways facilitated by opioids to produce both beneficial and adverse effects, thus driving research into the CB1R for pain management^4–6^.

G protein-gated, inwardly-rectifying potassium (GIRK) channels are potassium (K^+^) ion channels associated with opioid-induced analgesia^7^. Agonists at the CB1R produce a GIRK channel response by releasing the Gβγ_i_ subunit from the Gαβγ_i_ complex^8–10^. The Gβγ_i_ subunit binds to the GIRK channel, triggering an efflux of K^+^ ions, which hyperpolarizes the neuron^11,12^. This reaction decreases the formation of spontaneous action potentials and inhibits the release of excitatory neurotransmitters^13^. Following the initial G_i_ signaling cascade is the recruitment of β-arrestins 1 or 2 (β-arr1, β-arr2)^14^. In contrast to analgesic downstream effects of Gβγ_i_ signaling, β-arr2 mediates receptor desensitization and internalization, mechanisms closely associated with drug tolerance^15–18^. β-arr2 knockout mice exhibited decreased tolerance to antinociceptive effects and decreased CB1R desensitization and downregulation^19^.

This research compares molecular responses associated with antinociception and drug tolerance: GIRK channel activation and receptor internalization^19,20^. To measure GIRK channel responses and CB1R internalization, we used immortalized mouse pituitary gland cells, AtT20, stably transfected with a super-ecliptic pHluorin-human CB1R (SEPCB1) plasmid. AtT20 cells endogenously express heterotetramer GIRK1/2 channels and are reported to have neuronal-like properties^10,21,22^. The SEP construct is a green fluorescent protein (GFP) that, when tagged to a receptor, will emit a fluorescent signal when exposed to physiological pH, such as the extracellular space on the plasma membrane. The fluorescent signal decreases as the SEP-tagged receptor is exposed to increasingly acidic conditions, such as when a receptor is removed from the surface of the membrane and trafficked to the lysosome^22,23^. Using live AtT20-SEPCB1 cells, we measured cannabinoid-induced GIRK channel response and CB1R internalization in real-time using two assays. The GIRK channel assay used a membrane potential-sensitive dye, which captures the kinetic shift towards hyperpolarization due to the efflux of K^+^ ions^24,25^. CB1R internalization was measured by imaging AtT20-SEPCB1 pre- and post-cannabinoid administration over time. In addition, CB1R internalization was visualized by compiling images into time-lapse animations. With these two assays, we could compare a group of cannabinoids within a signaling pathway and across signaling pathways.

## Methods

### Cannabinoids

The following compounds were purchased from Cayman Chemical (Ann Arbor, MI, USA): (±) CP55,940, (+)-WIN 55,212-2 (mesylate), anandamide (AEA), Δ^9^-tetrahydrocannabinol (Δ^9^-THC), and SR141716. All controlled substances were purchased through the cannabis safety program at the University of Saskatchewan (HS-002).

### AtT20-SEPCB1 cell culture

The AtT20 pituitary cell line was obtained from ATCC (AtT-20/D16y-F2, CRL-1795) and grown in Dulbecco's Modified Eagle Medium (DMEM) with 10% fetal bovine serum (FBS) (ATCC Gibco—Manassas, VA) and 1% Penicillin–Streptomycin (Pen-Strep) (Cytiva Hyclone—Vancouver, BC) for the GIRK channel assay. For the CB1R internalization assay, cells were grown in FluoroBrite media (Gibco) with 10% FBS (ATCC Gibco—Manassas, VA), 1% Pen-Strep (Cytiva Hyclone—Vancouver, BC), 2% Glutamax (ATCC Gibco—Manassas, VA), and 10 mM HEPES (Sigma—Oakville, ON). AtT20 cells were stably transfected with lentivirus vectors containing the human cannabinoid type-1 receptor (CB1R) tagged at the *N*-terminus of the receptor with a super-ecliptic pHluorin (AtT20SEP-CB1) (from Dr. Andrew Irving, University College Dublin)^22^. The tagged-CB1R displays a response similar to the unmodified receptor^25^. Cells were plated in poly-l-lysine-coated wells of black 96-well plates (Greiner Bio-One—Monroe, NC) (50,000 cells per well). AtT20-SEPCB1 cells were stored in an incubator at 37 °C (5% O_2_/95% CO_2_) and used 24 h (CB1R Internationalization assay) or 72 h (GIRK channel assay) after plating. CB1R internalization occurred 24 h after plating because the measurements depended on selection of individual AtT20-SEPCB1 cells in comparison to the GIRK channel assay, which measures the overall movement of MP-sensitive fluorescent dye (MPSD) molecules on across the AtT20-SEPCB1 cell monolayer.

### GIRK channel assay and CB1R internalization assay

**Figure 1:**
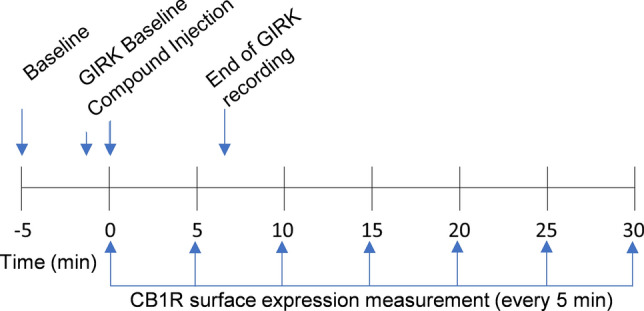
Experimental timeline for CB1R GIRK channel response quantification and internalization imaging. GIRK channel and CB1R internalization experiments were run separately but compared here for reference. Baseline images of AtT20-SEPCB1 cells were taken 5 min before exposure to a cannabinoid. Basal GIRK channel activity was recorded for 36 s prior to compound injection. The change in fluorescent signal, representing CB1R surface expression, was imaged at compound injection and every 5 min thereafter for 30 min. GIRK channel responses were recorded every 9 s for 240 s (i.e., 6 min) after compound exposure.

GIRK channel activation was monitored in the 96-well clear-bottom plates by recording cell membrane potential (MP) via fluorimetry as previously described^25,26^. For the MP measurements, the AtT20-SEPCB1 cells were incubated for 30 min in a buffer solution consisting of 132 mM NaCl, 5 mM KCl, 1 mM CaCl_2_, 1 mM MgCl_2_,5 mM dextrose, 5 mM HEPES, pH 7.4 (with NaOH), with MPSD (FLIPR Membrane Potential kit RED; MolecularDevices). Prior to the fluorescence measurements, the cells were loaded with MPSD in buffer solution (132 mM NaCl, 1 mM KCl, 1 mM CaCl_2_, 1 mM MgCl_2_,5 mM dextrose, 5 mM HEPES, pH 7.4 (with NaOH)as above) and incubated for an additional 5 min. Fluorescent signals were recorded using a SynergyHT Cytation microplate reader (Biotek) at 28 °C^25,26^. (±) CP55,940 and WIN55,212-2 were dissolved in DMSO at stock concentrations of 100 mM, AEA was dissolved in ethanol (as prepared by Cayman chemical), and Δ^9^-THC was dissolved in acetonitrile (as prepared by Cayman Chemical). The stock concentration was serially diluted for all cannabinoids in 1 mM KCl buffer solution containing the MPSD to create the working concentrations. The cannabinoids or control solution (20 μL) were injected into each well (total volume = 220 μL) at time zero. Data were collected at 9 s intervals from 36 s before compound addition until 240 s after compound addition (Fig. 1) at excitation and emission wavelengths of 520 and 560 nm, respectively.

### CB1R imaging

CB1R internalization was recorded in 96-well plates by imaging AtT20-SEPCB1R expression on the cell surface. pHluorin is a pH-sensitive green fluorescent protein whose cell surface fluorescence can be visualized at 525 nm. Because FBS increases background fluorescence and decreases image clarity, the FluoroBrite media used for cell culture was replaced with 100 μL FluoroBrite media containing 1% Pen-Strep, 2% Glutamax, 10 mM HEPES, and no FBS (Imaging media). Stock solutions of cannabinoids were diluted in imaging media to working concentrations. CB1R inverse agonist/antagonist, SR141716 was diluted in DMSO to a stock concentration of 3 mM, then diluted in imaging media. Images of AtT20-SEPCB1 cells were taken at 40 × using a BioTek Cytation 5 microplate reader (Agilent) at 28 °C with excitation and emission wavelengths 469 and 525 nm, respectively. Cannabinoids or control were pipetted into each well (10 μL) (total volume = 110 μL) at time zero. Z-stack images were comprised of 20, 1 μm sections collected in each well before (baseline) and after post-drug injection for 35 min divided into 5-min intervals.

### Imaging data analysis

Z-stacks were compressed into 1 image, representing the average fluorescent intensity per time point using BioTek Gen5 version 3.1 (Agilent, https://www.agilent.com/en/product/cell-analysis/cell-imaging-microscopy/cell-imaging-microscopy-software/biotek-gen5-software-for-imaging-microscopy-1623226). Further analysis of images was conducted using ImageJ/FIJI, 2023 version 2.15.1 (National Institute of Health ImageJ, https://imagej.net/software/fiji/). Each set of images were then aligned across all time points, and background was subtracted. Regions of interest (ROIs) were determined from cells in the baseline image (− 5 min), then the mean fluorescent intensities (F) were measured within the ROIs for each time point (− 5, 0 [time of compound addition], 5, 10, 15, 20, 25, and 30 min) (Fig. 1). For both GIRK channel response assays and CB1R internalization assays, change in fluorescent response (ΔF) post drug injection was normalized to the baseline fluorescent response values (F_o_), then the fluorescent response values from the control wells were subtracted: ΔF = ((F/F_o_) − scontrol).

### CB1R image and animation generation

Visualization of CB1R internalization was conducted using ImageJ/FIJI software version 2.15.1, as above. Z-stacks were compressed into 1 image per time point set to maximal fluorescence. Each set of images were then z-stacked and aligned across all time points (see representative videos in Supplementary files). Images were background subtracted, and then a FIRE look-up table (LUT) was applied to represent change in fluorescent intensity. These images were not used for data analysis.

### Statistical analysis

Data from GIRK channel assays were fit to a one-site exponential decay curve in GraphPad Prism (version 9.0) to estimate the rate of GIRK channel response (Supplementary Fig. 1). Data from GIRK channel assays were also analyzed using the Area Under the Curve function with default settings in GraphPad Prism. Peak F/F_0_ readings at 240 s for each cannabinoid were plotted against compound concentration (Supplementary Fig. 2). AUC and peak F/F_0_ data were then normalized to the (±)CP55,940 maximum and fit to the four-parameter, non-linear regression analysis in Graphpad Prism (v. 9.0): y = y_min_ + (y_max_ − y_min_/1 + 10^ ((LogEC_50_–Log Concentration), where EC_50_ is the concentration producing a 50% increase in the maximal response y_max_ (E_max_), and y_min_ is defined as a minimum fluorescent response. The same data analysis procedure was followed for CB1R internalization data using the one-site exponential decay curve for rate of internalization (Supplementary Fig. 3), AUC analysis (Supplementary Fig. 4) and subsequent concentration–response curve analyses. Data are presented as the mean ± standard error of the mean (S.E.M.). Statistical analyses were one- or two-way analysis of variance (ANOVA) followed by Tukey’s or Dunnett’s post-hoc tests (one-way ANOVA) or Bonferroni’s post-hoc test (two-way ANOVA), respectively and as indicated. P < 0.05 was considered statistically significant. Compound treatment replicates or individual cells are represented by n values, as indicated in figure legends.

## Results

### Cannabinoid-induced GIRK1/2 channel activation

**Figure 2:**
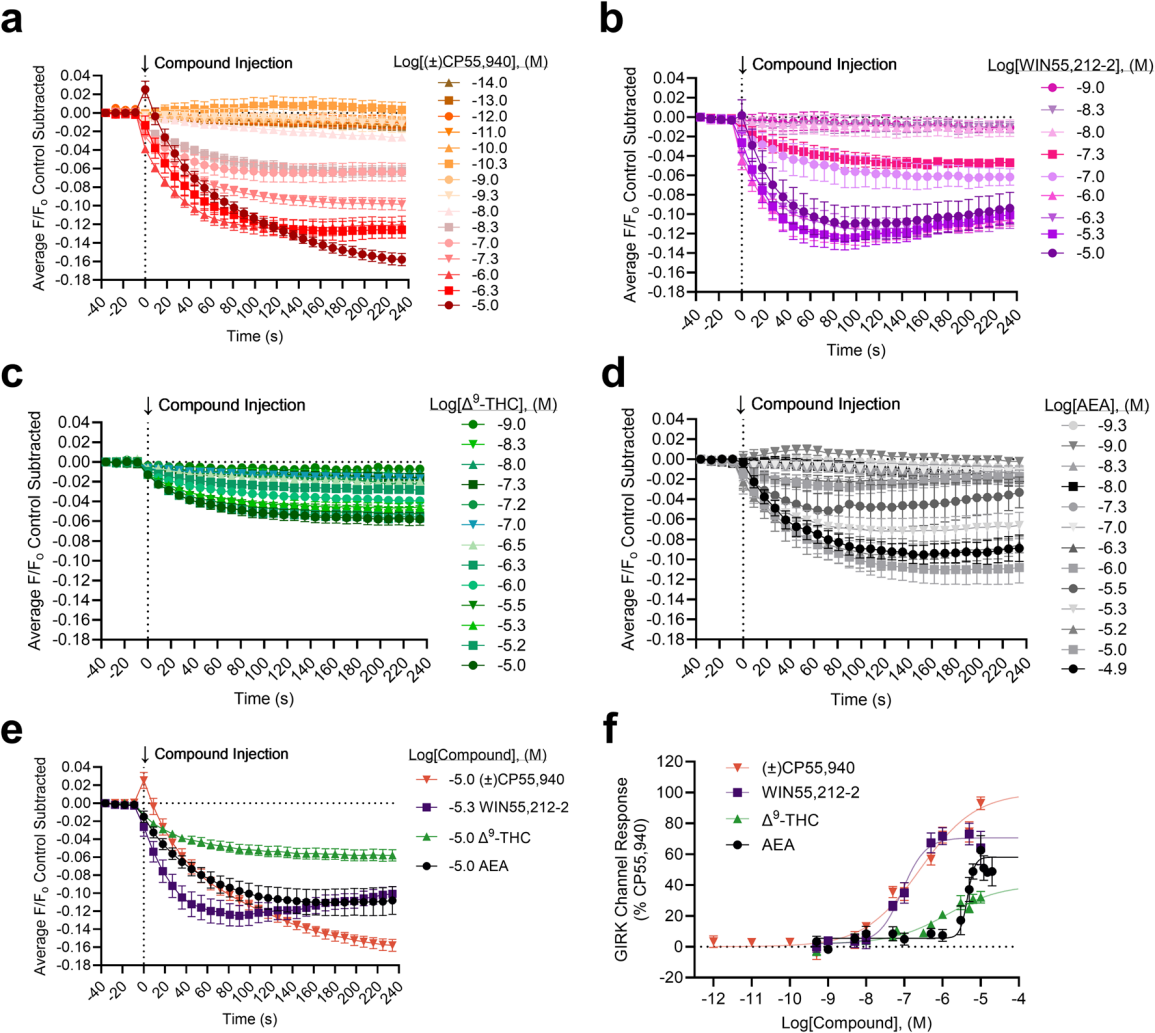
GIRK channel responses in AtT20 cells following cannabinoid treatment. AtT20 cells stably-expressing SEP-CB1R were treated with 10 fM to 10 μM of cannabinoids as indicated and GIRK channel response was measured continuously for 6 min (i.e., 240 s) with the mean time courses shown in panels (**a**)–(**d**). (**a**) (±)CP55,940 (10 fM—10 μM) *n* = 5–13. (**b**) WIN55,212-2 (0.5 nM to 10 μM) *n* = 4–5. (**c**) Δ^9^-THC (0.5 nM to 10 μM) *n* = 4–18. (**d**) AEA (0.5 nM to 20 μM) *n* = 3–16. (**e**) A comparison of the GIRK channel maximal responses for each cannabinoid from panels (**a**)–(**d**). [( ±)CP55,940 10 μM *n* = 13, WIN55,212–2 5 μM *n* = 5, Δ^9^-THC 10 μM *n* = 10, AEA 10 μM *n* = 16]. (**f**) Peak responses at 240 s for each compound were plotted against log[Compound], (M) and normalized to the maximal (±)CP55,940 response (i.e., 100%). Note that the 10 fM and 100 fM (±)CP55,940 concentrations were not included in the concentration–response curve. Data were fit to a four-parameter non-linear regression. Potency and efficacy data are presented in Table 1. All data are presented as mean ± S.E.M of *n* treatment replicates.

The kinetics and magnitude of the GIRK1/2 channel response depend on the cannabinoid bound to CB1R. Figure 2 illustrates the concentration-dependent change in membrane potential (MP) fluorescent response in the GIRK1/2 channel assay for (±)CP55,940 (Fig. 2a), WIN55,212-2 (Fig. 2b), Δ^9^-THC (Fig. 2c), and AEA (Fig. 2d) such that increasing concentrations evoke greater changes in the observed F/F_0_ values. Significant differences in the rate of change (i.e., slope) were not observed when responses were compared within each compound tested, suggesting no concentration-dependent change in GIRK response rate (Supplementary Fig. 1a–d). The maximum responses and corresponding concentrations are presented for comparison between the cannabinoids tested in Fig. 2e. When the rate of change was compared between these maximum responses—that is between 10 µM (±)CP55,940, 5 µM WIN55,212-2, 10 µM Δ^9^-THC, and 10 µM AEA—the rate of GIRK1/2 channel activation for 10 µM ( ±)CP55,940 was significantly slower than that of 5 µM WIN55,212-2, 10 µM Δ^9^-THC, or 10 µM AEA (Supplementary Fig. 1e). In addition, the rate of GIRK1/2 channel activation was significantly faster for 5 µM WIN55,212-2 compared to 10 µM AEA (Supplementary Fig. 1e). The peak GIRK1/2 channel response at 240 s was plotted against cannabinoid concentration and data were normalized to the maximum response observed for CP55,940; these data were then fit to a four-parameter non-linear regression to estimate cannabinoid potency and efficacy. The rank order potency of WIN55,212-2 > (±)CP55,940 > Δ^9^-THC > AEA with AEA being significantly less potent than (±)CP55,940 (Table 1, Fig. 2f). The rank order of efficacy was (±)CP55,940 > WIN55,212-2 > AEA > Δ^9^-THC, with Δ^9^-THC and AEA being significantly less efficacious than (±)CP55,940 (Table 1, Fig. 2f). However, we observed that the 10 µM (±)CP55,940 response was notably elevated compared to 1 µM (±)CP55,940 and was likely driving the efficacy calculation for (±)CP55,940 (Fig. 2f). Therefore, to further determine whether differences in GIRK1/2 channel maximum response affected the rank order potency or efficacy of cannabinoids, the AUC was calculated for each GIRK1/2 channel response and graphed against each cannabinoid concentration (Supplementary Fig. 2). These data were fit to a four-parameter non-linear regression and in this analysis although the rank order efficacy was not different from our calculations using peak GIRK channel response at 240 s, WIN55,212–2 and (±)CP55,940 have highly similar E_max_ values (95% versus 100%, respectively) (Supplementary Fig. 2). Therefore WIN55,212-2 and (±)CP55,940 do not likely differ in efficacy in this assay.

**Table 1:** GIRK channel responses and CB1R internalization in AtT20 cells following cannabinoid treatment. AtT20 cells stably-expressing SEP-CB1R were treated with 10 fM – 10 μM of cannabinoids as indicated and GIRK channel response was measured continuously for 6 min (i.e., 240 s) with the mean time courses shown in figure 2a-e, or CB1R internalization was measured at 5 min intervals for 30 min with the mean time courses shown in figure 4a-e. Here, peak responses at 240 s (GIRK) and 30 min (internalization) for each compound were plotted against log[Compound], (M) and normalized to the maximal (±)CP55,940 response (i.e., 100%). Data were fit to a four-parameter non-linear regression (Fig. 2f, 4f) to estimate potency and efficacy. All data are presented as mean ± S.E.M. *p<0.05, **p<0.01, ****p<0.0001 compared to (±)CP55,940 as determined by one-way ANOVA within assay followed by Dunnett's post-hoc test.

Compound	Peak GIRK response		CB1R internalization	
	pEC_50_ ± S.E.M (nM)	E_max_ (%) ± S.E.M	pEC_50_ ± S.E.M (nM)	E_max_ (%) ± S.E.M
(±)CP55,940	6.6 ± 0.15 (250)	100 ± 7.4	7.3 ± 0.49 (54)	72 ± 7.2
WIN55,212-2	7.1 ± 0.10 (88)	71 ± 3.7	6.7 ± 0.40 (200)	85 ± 7.2
Δ^9^-THC	5.9 ± 0.55 (1300)	40 ± 14****	5.6 ± 0.48 (2700)	49 ± 7.8
AEA	5.3 ± 0.05 (4000)***	58 ± 4.9***	5.7 ± 0.81 (1900)	86 ± 21

### CB1R internalization can be imaged and quantified using AtT20-SEPCB1 cells

#### Establishing the CB1R internalization assay

**Figure 3:**
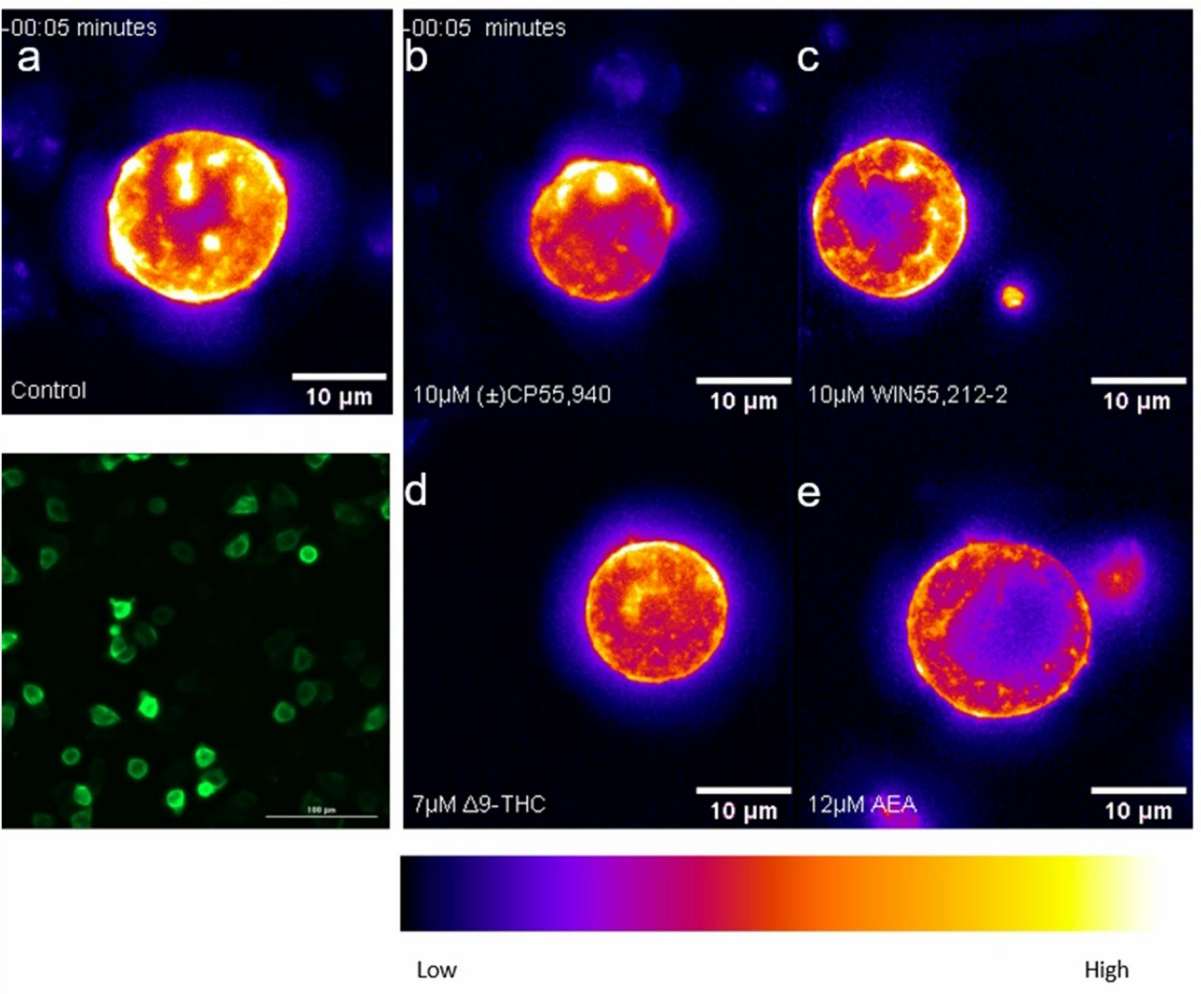
CB1R internalization in AtT20 cells following cannabinoid treatment. AtT20 cells stably-expressing SEP-CB1R were treated with 0.1 nM to 12 μM of cannabinoids as indicated and CB1R internalization was measured at 5 min intervals. Representative video montages are presented here for vehicle (**a**), 10 μM (±)CP55,940 (**b**), 1 μM WIN55,212-2 (**c**), 7 μM Δ^9^-THC (**d**), and 12 μM AEA (**e**) in false colour generated using Fiji. (**a**, lower left panel) A true-colour baseline confocal image is presented at ×40 magnification. The image is composed of the average fluorescent signal generated from 20, 1 μm images in a compressed z-stack. Quantification of internalization experiments is presented in Fig. 4.

The CB1R internalization experiments followed the GIRK1/2 channel assay protocol modified for imaging. AtT20-SEPCB1 cells were cultured in clear-bottom, black-walled, 96-well plates, with two wells being vehicle controls and the rest treated with the compounds. Cells were recorded at 40 × magnification at 5 min intervals following treatment with vehicle, (±)CP55,940, WIN55,212-2, Δ^9^-THC, or AEA (see Supplementary video files for Fig. 3), and a false-color heat map was applied to images to visualize SEP-CB1R in video montages (Fig. 3a–e). Most AtT20 cells expressed GFP labeling, thus confirming stable transfection with the SEPCB1 construct (Fig. 3a [lower panel]).

#### Synthetic cannabinoids potently induce CB1R internalization

**Figure 4:**
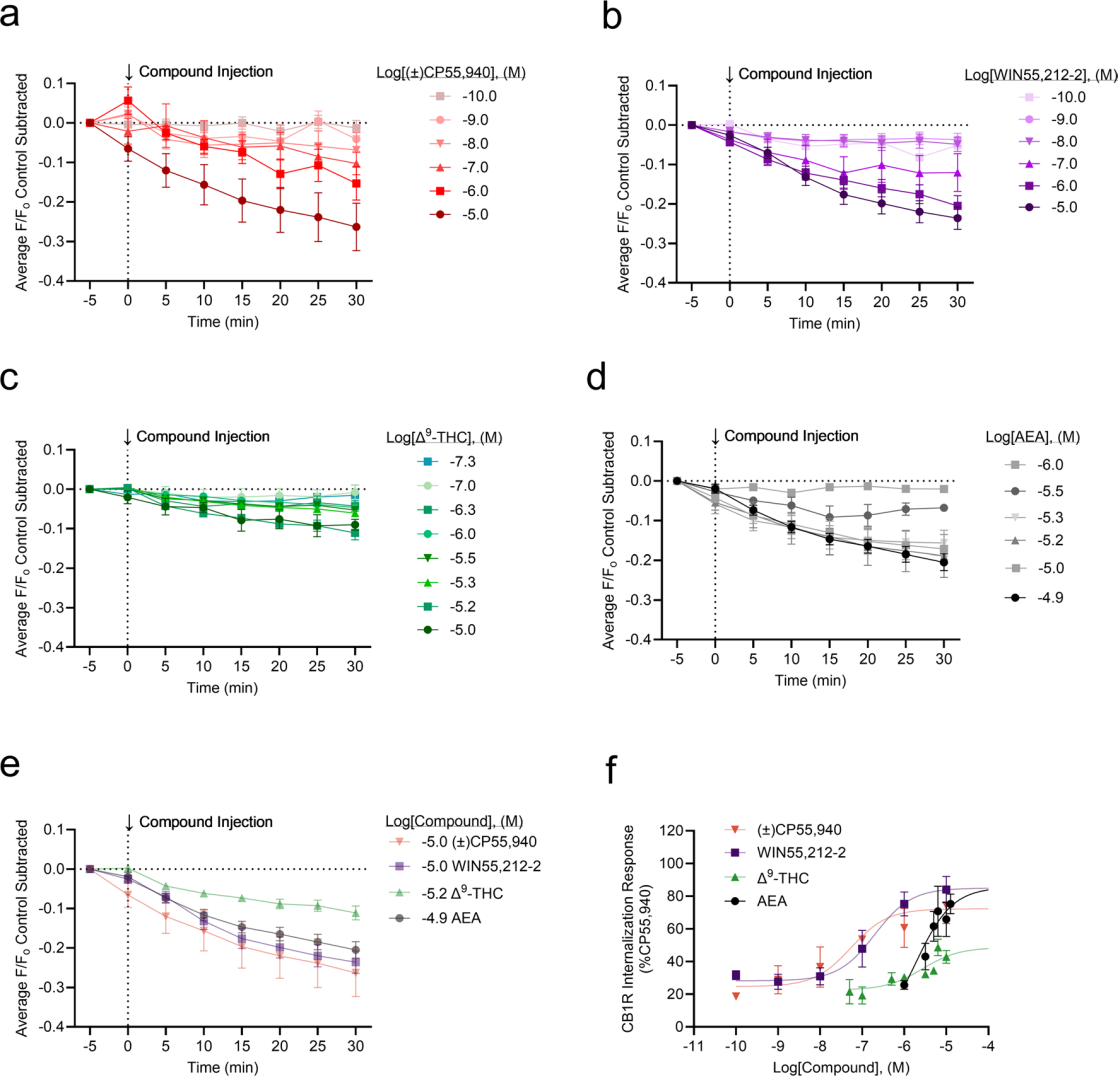
CB1R internalization in AtT20 cells following cannabinoid treatment. AtT20 cells stably-expressing SEP-CB1R were treated with 0.1 nM to 10 μM of cannabinoids as indicated and CB1R internalization was measured at 5 min intervals with the mean time courses shown in panels (**a**)–(**d**). (**a**) (±)CP55,940 (0.1 nM to 10 μM) *n* = 2–7. (**b**) WIN55,212-2 (0.1 nM to 10 μM) *n* = 4–47. (**c**) Δ^9^-THC (50 nM to 10 μM) *n* = 2–27. (**d**) AEA (1–12 μM) *n* = 4–11. (**e**) A comparison of the CB1R internalization maximal responses for each cannabinoid from panels (**a**)–(**d**). [(±)CP55,940 10 μM *n* = 7, WIN55,212-2 10 μM *n* = 32, Δ^9^-THC 10 μM *n* = 22, AEA 12 μM *n* = 9]. (**f**) Peak responses at 30 min for each compound were plotted against log[Compound], (M) and normalized to the maximal (±)CP55,940 response (i.e., 100%). Data were fit to a four-parameter non-linear regression with the Hill Slope constrained to 1. Potency and efficacy data are presented in Table 1. All data are presented as mean ± S.E.M. of *n* treated cells.

**Figure 5:**
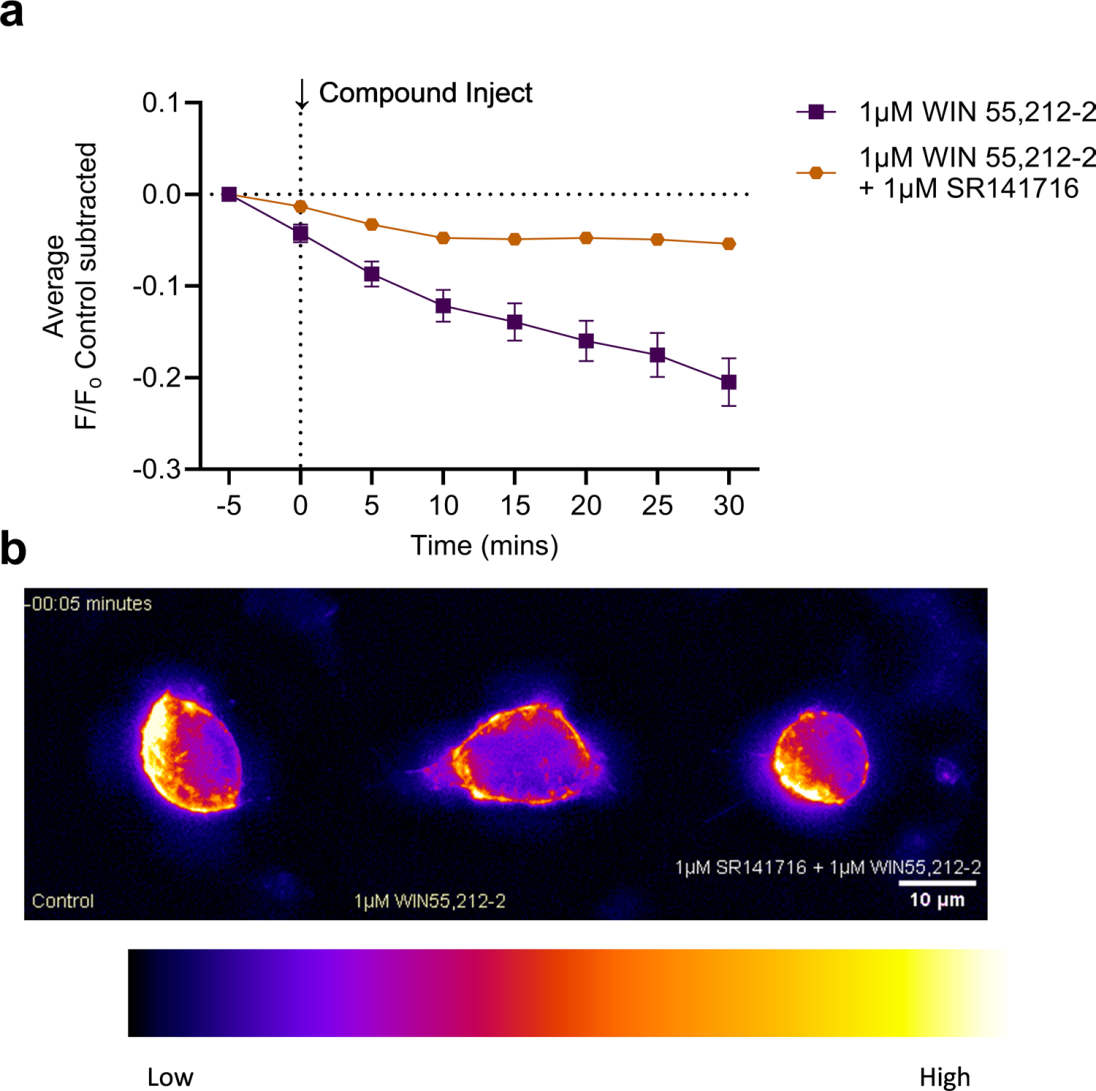
CB1R internalization in AtT20 cells following cannabinoid treatment. AtT20 cells stably-expressing SEP-CB1R were treated with 1 μM WIN55,212-2 with or without 1 μM of the CB1R inverse agonist SR141716A as indicated and CB1R internalization was measured at 5 min intervals with the mean time courses shown. 1 μM WIN55,212-2 *n* = 47, 1 μM WIN55,212-2 + 1 μMSR141716A *n* = 57. WIN55,212-2 are the same as those presented in Fig. 4. All data are presented as mean ± S.E.M. of *n* treated cells.

CB1R internalization was imaged and measured for (±)CP55,940, WIN55,212-2, Δ^9^-THC, and AEA (Fig. 4a–d). WIN55,212-2 produced the greatest CB1R internalization, whereas Δ^9^-THC produced the least (Fig. 4c,e). As was done for the GIRK channel assay, peak CB1R internalization at 30 min was plotted against concentration, and rank order potency was determined to be WIN55,212–2 > (±)CP55, 940 > Δ^9^-THC > AEA (Fig. 4f, Table 1). The rank order efficacy was (±)CP55, 940 > WIN55,212-2 > AEA > Δ^9^-THC (Fig. 4f, Table). These rank orders of potency and efficacy were the same as observations made in the GIRK channel assay. However, no statistically significant differences were detected between (±)CP55, 940 and other compounds in the CB1R internalization assay. In general, cannabinoid potency was less in the CB1R internalization assay than in the GIRK assay, although these differences were not statistically significant (as determined by two-way ANOVAs followed by Bonferroni’s post-hoc test). Co-treatment of cells with 1 µM WIN55,212-2 and 1 µM SR141716 reduced CB1R internalization, indicating the quantification approach used was measuring CB1R trafficking (see Supplementary video files for Fig. 5; Fig. 5). SR141716 was not assessed alone in these experiments and therefore the reason that this antagonist did not fully reverse WIN55,212-2 mediated CB1R internalization is not clear. Further assessment of CB1R trafficking in response to antagonists and inverse agonists with this model system is needed. As with the GIRK channel response, the kinetics of CB1R internalization may depend on the cannabinoid bound to CB1R. No significant change in the slope was observed when responses were compared within each compound tested (Supplementary Fig. 3a–d). When the slope was compared between these maximum responses, the rate of CB1R internalization was not significantly different between cannabinoids (Supplementary Fig. 3e). The AUC was calculated for each CB1R internalization response and graphed against each cannabinoid concentration (Supplementary Fig. 4). These data were fit to a four-parameter non-linear regression. In this analysis, the rank order potency and efficacy were not different from our calculations using peak CB1R internalization response at 30 min (Supplementary Fig. 4).

## Discussion

Studies into biased signaling and receptor-ligand binding highlight the diversity of cannabinoid-CB1R molecular signaling^27–29^. This research targets the GIRK1/2 channel and CB1R internalization responses of four cannabinoids: (±)CP55,940, WIN55,212-2, AEA, and Δ^9^-THC. In this study, the synthetic cannabinoids (±)CP55,950 and WIN55,212-2 were more potent and efficacious at stimulating a GIRK1/2 channel response than AEA and Δ^9^-THC, aligning with previous research^25,30^. Specifically, the trace of (±)CP55,940's GIRK1/2 response significantly differed from the other cannabinoids, suggesting different GIRK1/2 channel kinetics (Fig. 2e and Supplementary Fig. 1e). Of note, GIRK channel responses to these cannabinoids were not tested in cells lacking CB1R; therefore, non-cannabinoid receptor effects on GIRK channels by these ligands can not be ruled out in our findings. Synthetic cannabinoids have been shown to form stronger interactions within the CB1R binding pocket, which may induce conformational changes that promote G_i_ signaling^28,31,32^. Importantly, this study focused on AEA and did not include 2-arachidonoylglycerol, which has been described elsewhere as more potent and efficacious than AEA^23^; future studies should compare these two endocannabinoids for differential responses in these assays.

Phosphorylation of the CB1R by specific G protein-coupled receptor kinases (GRKs) aids in the recruitment of β-arr2, which then blocks the reassembly of the Gαβγ_i_ complex, leading to receptor desensitization and internalization^23,33,34^. Supporting the link between β-arrestins 1 and 2 and CB1R internalization is research by Flores-Otero et al., who found WIN55,212-2 recruits β-arr in parallel with CB1R internalization^15^. Research has also demonstrated that the CB1R internalization response varies depending on the cannabinoid^35^. In this study, the synthetic cannabinoids ranked higher in potency than AEA and Δ^9^-THC. In line with this, Δ^9^-THC binds to the CB1R in such a way that it forms a less stable active confirmation than synthetic cannabinoid receptor agonists^28,36^. Interestingly, AEA was more effective at inducing CB1R internalization compared to (±)CP55, 940, albeit this difference was not statistically significant and with lower potency. Similar to Δ^9^-THC, AEA is proposed to have unstable interactions with residues promoting CB1R-G_i_ signaling as opposed to CP55,940, which produce confirmation changes favorable to G_i_ signaling^28,29^. Sites implicated for β-arr2 recruitment and CB1R internalization include the c-terminus and transmembrane helices 2 (TMH2) and 7 (TMH7), whereas site such as α5 and intracellular loop 2 (ICL2) are important for CB1R-G_i_^23,28,37^.

While different sites on the CB1R proposed for β-arr2 and Gα_i_ functions exist, research has shown that GIRK1/2 channel function and CB1R internalization are mediated by the same amino acid residues on the CB1R^38^. In AtT20 cells, a D164N mutation on TM2 inhibited CB1R internalization and potentiation of GIRK channel current^34,38^. When comparing a cannabinoid's GIRK1/2 channel assays and CB1R internalization results, no significant differences were found between potency and efficacy. These results suggest that when a cannabinoid binds to the CB1R, the effects of the Gβγ_i_ signaling and β-arr2 recruitment are balanced. It is worth noting that within the GIRK1/2 channel assay, there were significant differences between the GIRK1/2 channel potency and efficacy of (±)CP55,940 compared to AEA and Δ^9^-THC; however, when repeated with CB1R internalization, no significant differences were observed. We considered that the significant differences found in the GIRK1/2 channel assay did not translate to the CB1R internalization assay because we measured peak responses at different time points. The first wave of CB1R intercellular signaling occurs rapidly and is primarily G_i_-driven, whereas peak β-arr2 occurs approximately 20 min later^14,16^. Peak GIRK1/2 channel and CB1R internalization responses were determined within the appropriate time frames; therefore, the lack of significant differences in the CB1R internalization assay is unlikely due to its peak response being missed at an earlier time point. This may be due to variability in the internalization assay, such that the error was too large to detect a statistically significant difference. One potential limitation of these data is that acidification of the extracellular environment could have influenced measurements of fluorescence with the SEPCB1construct. This could be assessed in future studies by alkalinization at the end of experiments; however, video montages support the occurrence of internalization. Theoretically, if the same location on the CB1R mediates GIRK1/2 channel activation and CB1R internalization, then significant differences in the GIRK1/2 channel assay would extend to the CB1R internalization. This discrepancy highlights the need for further research, specifically, kinetic measurements of β-arr2 recruitment to clarify the precise signaling mechanisms involved in CB1R internalization.

In summary, GIRK channels and receptor internalization are two molecular responses central to CB1R signaling. These mechanisms play a crucial role in determining the physiological response to cannabinoids, which are presented as options for pain relief and, therefore, should be further investigated.

### Supplementary Information

Supplementary Figures.

Supplementary Video 1.

Supplementary Video 2.

## Data Availability

Supplemental analyses are presented in the supplemental data for this manuscript. All datasets generated and/or analyzed during the current study are accessible through the Dryad repository at 10.5061/dryad.r4xgxd2nz.
