# Supplementary material for: Comparing CB1 receptor GIRK channel responses to receptor internalization using a kinetic imaging assay
Source: Sci Rep. 2024 Aug 7;14:18314. doi: 10.1038/s41598-024-68451-2 (PMC11306342; doi:10.1038/s41598-024-68451-2)
Supplement: Supplementary file 1 — Supplementary Figures. [file 41598_2024_68451_MOESM1_ESM.docx]

**Supplementary Data for:** **COMPARING CB1 RECEPTOR GIRK CHANNEL RESPONSES TO RECEPTOR INTERNALIZATION USING A KINETIC IMAGING ASSAY**

**Haley K. Andersen^1^, Duncan G.Vardakas^1^, Julie A. Lamothe^1^, Tannis E.A. Perault^1^, Kenneth B. Walsh^2^, Robert B. Laprairie^1,3^***





**Supplementary Figure 1**: *GIRK channel responses in AtT20 cells following cannabinoid treatment*. AtT20 cells stably-expressing SEP-CB1R were treated with 10 fM – 10 μM of cannabinoids as indicated and GIRK channel response was measured continuously for 6 min (i.e., 240 s). The rate of response (k, in units s^-1)^ was estimated by fitting data from individual experiments to a one-phase exponential decay curve in GraphPad Prism (v. 9.0). **a)** (±)CP55,940 (10 fM – 10 μM) *n=*5-13. **b)** WIN55,212-2 (0.5 nM - 10 μM) *n*=4-5. c) Δ^9^-THC (0.5 nM - 10 μM) *n*=4-18. d) AEA (0.5 nM – 20 μM) *n*=3-16. e) A comparison of the rate of GIRK channel maximal responses for each cannabinoid from panels a-d. [(±)CP55,940 10 μM *n*=13, WIN55,212-2 5 μM *n*=5, Δ^9^-THC 10 μM *n*=10, AEA 10 μM *n*=16]. These data correspond to data presented in Figure 2 and Table 1. All data are presented as mean ± S.E.M. with individual data points of *n* treatment replicates. ****p<0.0001, ***p<0.001, **p<0.01 compared to (±)CP55,940; ^p<0.05 compared to WIN55,212-2 as determined by one-way ANOVA followed by Tukey’s post-hoc test.


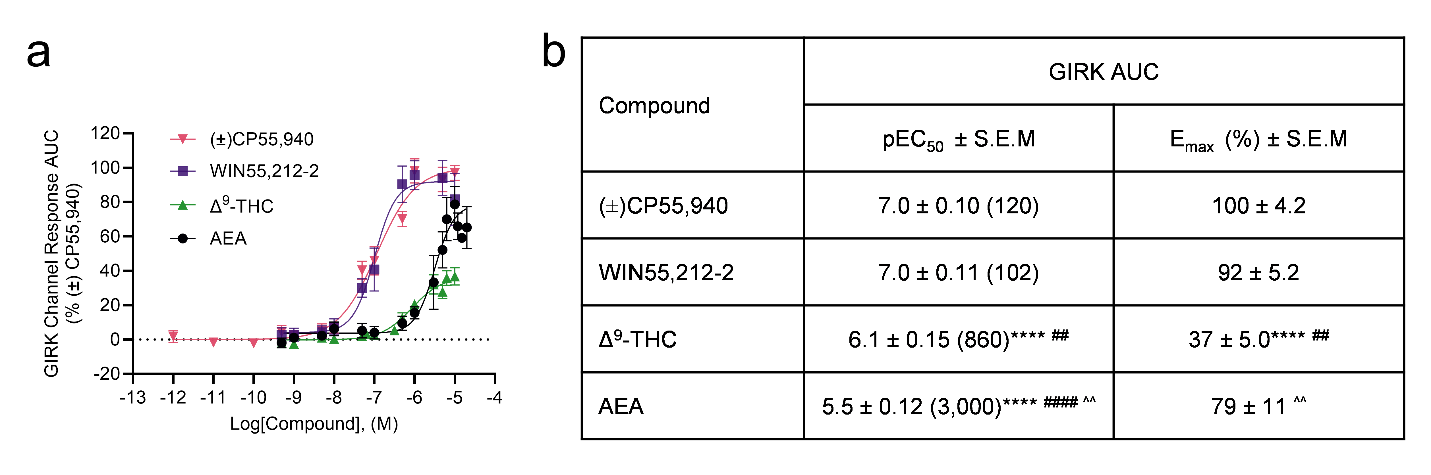


**Supplementary Figure 2:** *GIRK channel responses in AtT20 cells following cannabinoid treatment*. AtT20 cells stably-expressing SEP-CB1R were treated with 1 pM – 15 μM of cannabinoids as indicated and GIRK channel response was measured continuously for 6 min (i.e., 240 s) with corresponding data presented in Figure 2 [(±)CP55,940 10μM *n*=5-13, WIN55,212-2 5μM *n*=4-5, Δ^9^-THC 10 μM *n*=4-18, AEA 10 μM *n*=3-16]. **a)** Total AUC from curves presented in Fig. 2a-d was calculated and plotted here against log[Compound], (M) and normalized to the maximal (±)CP55,940 response (i.e., 100%). Data were fit to a four-parameter non-linear regression. Potency and efficacy data are presented in **b**. All data are presented as mean ± S.E.M. of *n* treatment replicates. ****p<0.0001, compared to (±)CP55,940; ^####^p<0.0001, ^##^p<0.01 compared to WIN55,212-2; ^^p<0.01 compared to Δ^9^-THC as determined by one-way ANOVA followed by Tukey’s post-hoc test.


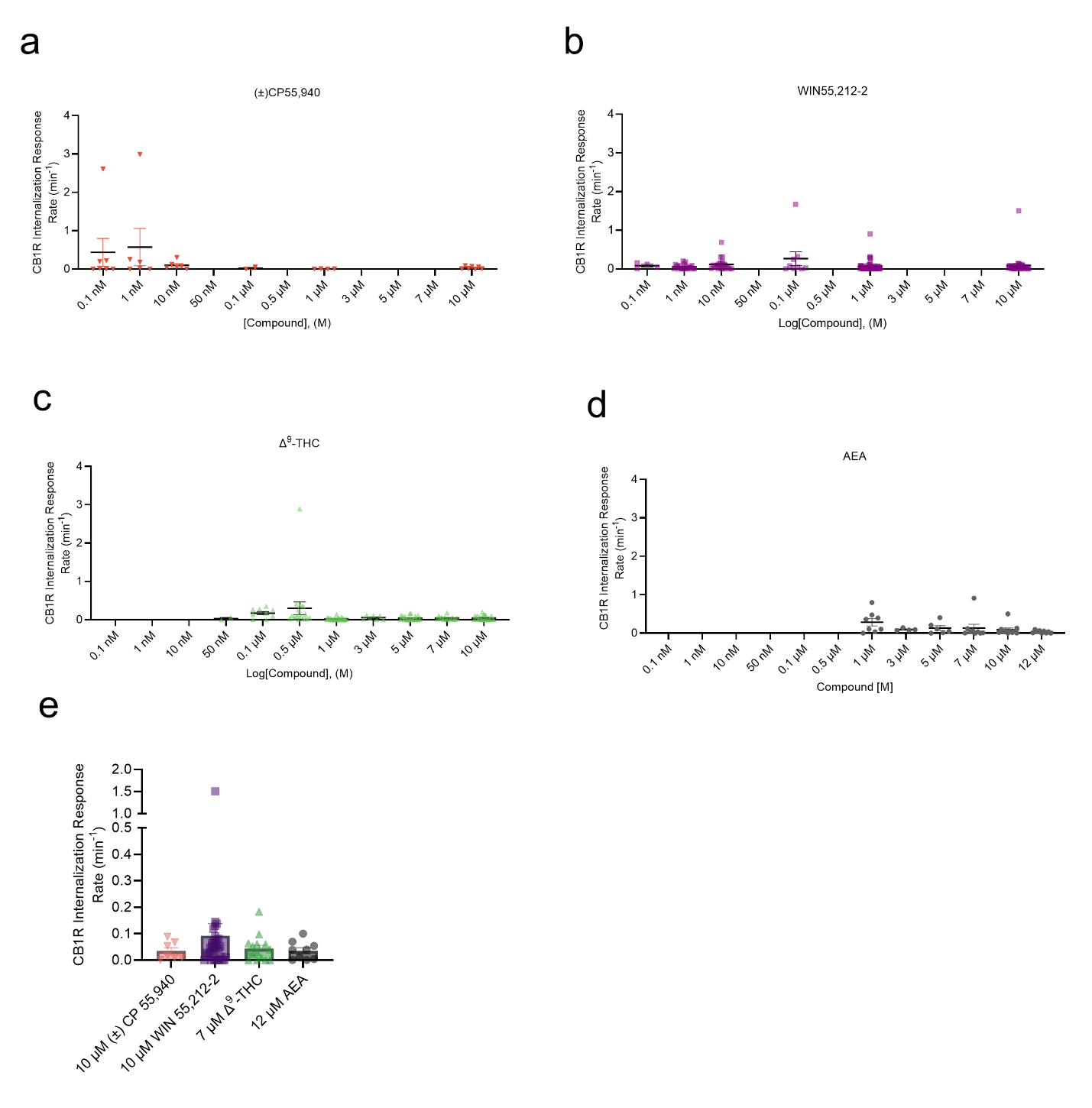


**Supplementary Figure 3:** *CB1R internalization in AtT20 cells following cannabinoid treatment*. AtT20 cells stably-expressing SEP-CB1R were treated with 0.1 nM – 10 μM of cannabinoids as indicated and CB1R internalization was measured at 5 min intervals for 30 min. The rate of response (k, in units min^-1^) was estimated by fitting data from individual experiments to a one-phase exponential decay curve in GraphPad Prism (v. 9.0). **a)** (±)CP55,940 (0.1 nM – 10 μM) *n=*2-7. **b)** WIN55,212-2 (0.1 nM - 10 μM) *n*=4-47. **c)** Δ^9^-THC (50 nM - 10 μM) *n*=2-27. **d)** AEA (1 μ M – 12 μM) *n*=4-11. **e)** A comparison of the rate of CB1R internalization maximal responses for each cannabinoid from panels **a-d**. [(±)CP55,940 10 μM *n*=7, WIN55,212-2 10 μM *n*=32, Δ^9^-THC 7 μM *n*=15, AEA 12 μM *n*=9]. These data correspond to data presented in Figure 4 and Table 1. All data are presented as mean ± S.E.M with individual data points for of *n* treated cells. Note the scale of the y-axis in panel **e** is different.


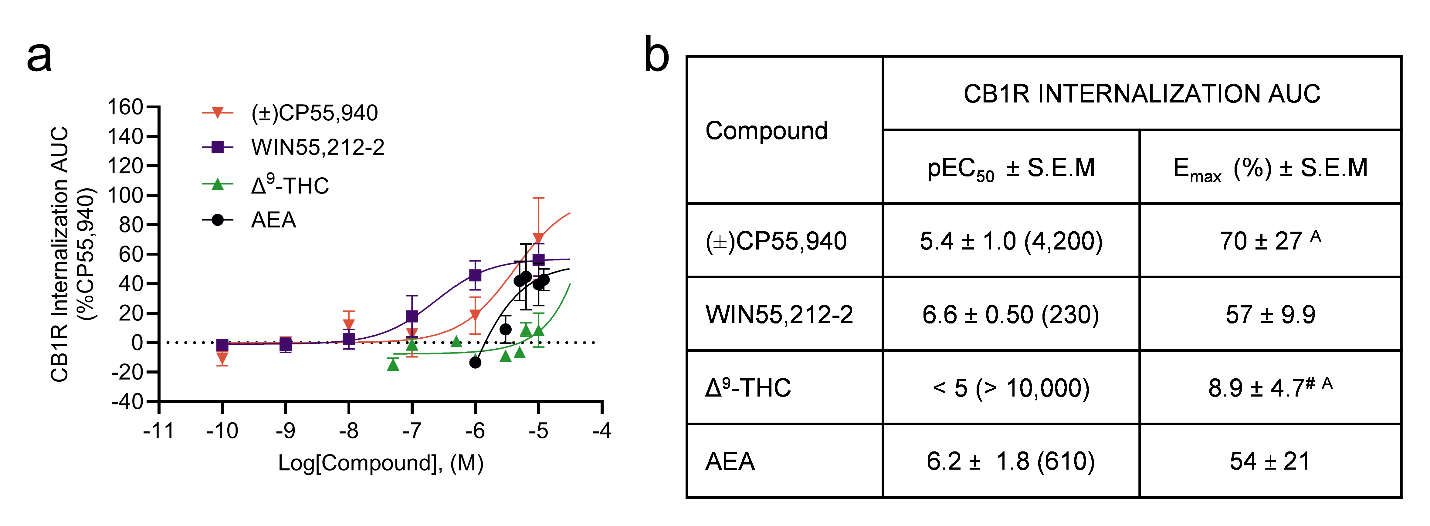


**Supplementary Figure 4:** *CB1R internalization in AtT20 cells following cannabinoid treatment*. AtT20 cells stably-expressing SEP-CB1R were treated with 0.1 nM – 12 μM of cannabinoids as indicated and CB1R internalization was measured at 5 min intervals over 30 min with corresponding data presented in Figure 4 **)** (±)CP55,940 (0.1 nM – 10 μM) *n=*2-7, WIN55,212-2 (0.1 nM - 10 μM) *n*=4-47, Δ^9^-THC (50 nM - 10 μM) *n*=2-27, AEA (1 μ M – 12 μM) *n*=4-11]. **a)** Total AUC from curves presented in Fig. 4a-d was calculated and plotted here against log[Compound], (M) and normalized to the maximal (±)CP55,940 response (i.e., 100%). Data were fit to a four-parameter non-linear regression with the Hill Slope constrained to 1. Potency and efficacy data are presented in **b**. All data are presented as mean ± S.E.M. of *n* treated cells. ^#^p<0.05 compared to WIN55,212-2 as determined by one-way ANOVA followed by Tukey’s post-hoc test.; **^A^**Data are presented as highest observed value ± S.E.M. due to poor curve fit.
